# Role of Bio-Based and Fossil-Based Reactive Diluents in Epoxy Coatings with Amine and Phenalkamine Crosslinker

**DOI:** 10.3390/polym15193856

**Published:** 2023-09-22

**Authors:** Pieter Samyn, Joey Bosmans, Patrick Cosemans

**Affiliations:** Department of Innovations in Circular Economy and Renewable Materials, SIRRIS, 3001 Leuven, Belgium; joey.bosmans@sirris.be (J.B.); patrick.cosemans@sirris.be (P.C.)

**Keywords:** epoxy, diluent, bio-based, amine curing, coating

## Abstract

The properties of epoxy can be adapted depending on the selection of bio-based diluents and crosslinkers to balance the appropriate viscosity for processing and the resulting mechanical properties for coating applications. This work presents a comprehensive study on the structure–property relationships for epoxy coatings with various diluents of mono-, di-, and bio-based trifunctional glycidyl ethers or bio-based epoxidized soybean oil added in appropriate concentration ranges, in combination with a traditional fossil-based amine or bio-based phenalkamine crosslinker. The viscosity of epoxy resins was already reduced for diluents with simple linear molecular configurations at low concentrations, while higher concentrations of more complex multifunctional diluents were needed for a similar viscosity reduction. The curing kinetics were evaluated through the fitting of data from differential scanning calorimetry to an Arrhenius equation, yielding the lowest activation energies for difunctional diluents in parallel with a balance between viscosity and reactivity. While the variations in curing kinetics with a change in diluent were minor, the phenalkamine crosslinkers resulted in a stronger decrease in activation energy. For cured epoxy resins, the glass transition temperature was determined as an intrinsic parameter that was further related to the mechanical coating performance. Considerable effects of the diluents on coating properties were investigated, mostly showing a reduction in abrasive wear for trifunctional diluents in parallel with the variations in hardness and ductility. The high hydrophobicity for coatings with diluents remained after wear and provided good protection. In conclusion, the coating performance could be related to the intrinsic mechanical properties independently of the fossil- or bio-based origin of diluents and crosslinkers, while additional lubricating properties are presented for vegetable oil diluents.

## 1. Introduction

Epoxy resins are frequently used in various sectors of industrial construction and the manufacturing of adhesives, coatings and paints, composites, primers and sealants, flooring, and tooling [1]. Due to its exceptional properties, bio-based epoxy materials have recently been introduced in advanced applications for aeronautics [2], aerospace engineering [3], electronic materials, and biomedical devices [4]. The long-term mechanical performance, chemical stability, anticorrosion properties [5], and thermal resistance [6] of bio-based epoxy are primary selection criteria, which can be tuned depending on the selection of the composition of the respective components, including epoxy resin or prepolymers [7], hardeners or crosslinkers [8], and diluents or fillers [9]. With an increasing need for sustainable sourcing of coating ingredients, the exploration of renewable polymers is urgent. While one-by-one replacement of fossil-based polymers often does not yield the best results, the coating system should be better redesigned to fully exploit the inherent features of its bio-based ingredients. The effects of bio-based phenalkamine versus traditional amine crosslinkers for epoxy coatings were evaluated in our previous work [10], while the incorporation of bio-based diluents may further increase the bio-based content of epoxy coatings, as discussed in this study.

The high reactivity of the epoxide ring in combination with crosslinking agents results in the formation of a three-dimensional polymer network with properties that depend on the molecular structure of the reactants and/or co-reactants. The structure provides thermoset properties, where the mechanical properties depend on the crosslinking density and the flexibility of the polymer segments between the anchoring points. The visco-elastic properties can be controlled by a combination of curing conditions (temperature, time) [11] and the composition of a partially bio-based epoxy resin [12] or bio-based epoxy prepolymers [7], offering a combined toolbox to create versatile end-user properties. In particular, the toughness of partially bio-based epoxy resins was improved after the optimization of the curing and post-curing conditions [13]. At the same time, the fluent processing of bio-based epoxy in coatings or composites needs lower viscosity to improve flow properties [14]. The addition of proper diluents aids in controlling the rheology and reducing the viscosity [15,16], improves the wettability of pigments and fillers, increases the pot-life and gelation time, and may limit the effects of curing shrinkage [17]. In particular, the rheological profile of a three-component system with epoxy, diluent, and amine crosslinker was detailed [18], concluding that the solid and pseudoplastic behavior introduced through the diamine is diminished in the presence of a diluent and the system tends more toward displaying Newtonian behavior. As such, the low molecular weight diluents take up the role of solvents and allow for smaller concentrations of volatile organic compounds [19]. Traditional petroleum-based diluents for epoxy should be used with care since allergic reactions toward phenyl glycidyl ether, 1,4-butanediol diglycidyl ether, and *p*-tert-butylphenyl glycidyl ether were identified [20]. Therefore, alternative bio-based diluents for epoxy resin were developed from furan [21], vegetable oils [22], cardanol [23], or glycerol [24]. The latter mainly allows for a higher functionality of reactive groups [25], resulting in more rigid mechanical properties.

The reactive diluents participate in the crosslinking process of epoxy resins and are covalently bonded into the polymer network; hence, they do not migrate, in contrast with traditional solvents or non-reactive diluents. The reactive diluents contain reactive epoxy groups organized in aliphatic or aromatic glycidyl ethers of alcohols and alkylphenols, while the non-reactive diluents typically include aromatic hydrocarbons, such as toluene, xylene, phthalates, styrene, or phenolic compounds. Although the non-reactive diluents are expected to not decrease the reactivity of the epoxy and can be added in relatively large concentrations, it was observed that the mechanical properties of cured epoxy systems were reduced after adding toluene [26]. Alternatively, the reactive diluents are more efficiently used in relatively small amounts and their reactivity is determined by the number of reactive sites (mono-, di-, or trifunctional). However, the influence of reactive diluents highly depends on the type of resin or diluent. Their effect is not uniformly subject to the mechanical characteristics [27], with a general improvement in modulus and strength and pendant compressive properties. The monofunctional glycidyl ether causes a significant drop in thermal stability and glass transition temperatures but with little effect on the glassy modulus [28]. The use of difunctional reactive diluents was reported as the most advantageous since they enhanced the curing process to achieve a high degree of crosslinking [29]. While comparing the performance of diglycidyl ether with other toughening agents, it was concluded that tensile strength and strain at break values are higher for the formulations with diluent compared with resins with a toughening agent [30]. In general, the properties of epoxy/diluent systems can be examined in relation to the crosslinking density and chain flexibility that both increase with the amount of diluent, while a drop in elasticity was associated with secondary relaxations [31]. Alternatively, the role of natural oils (e.g., epoxidized soybean oil and rapeseed oil) as reactive diluents indicated that viscosity reduction was comparable with commercial grade active diluents, but mechanical strength and thermal stability reduced due to the plasticizing effects of the oil [32]. The reactive bio-based diluents for epoxy are traditionally synthesized from soybean oil because of its high reactivity, including siloxane, allyl ether, and fluorine functionalization [33]. The role of epoxidized soybean oil with amine hardener on the curing kinetics of epoxy systems was studied and indicated similar behavior compared with conventional resins [34]. Also, epoxidized cardanol oil is a favored diluent for lowering the curing temperature without affecting the final degree of curing [35]. The oil diluents were typically applied in higher concentrations ranging from 20 wt.-% [36] up to 60 wt.-% [37], where the flexible epoxy materials with fast elastic recovery were obtained with low water absorption and high chemical resistance. The toughening effect of epoxidized vegetable oils in combination with a bio-based crosslinker was demonstrated to overcome the brittleness of bio-based epoxies [38]. However, the influences of diluents on mechanical properties may be contradictory when comparing different studies, either improving mechanical properties (strength, deformation) [39] or decreasing the modulus and ultimate strength while improving ductility [40]. The latter definitely depends on the combination of specific diluents with given crosslinkers, concentrations, and compatibility with the base resin.

In this work, epoxy coatings were formulated with a range of diluents, including those with various origins, functionalities, and suitable concentrations, to evaluate their effects on the processing and mechanical performance of coatings, while their compatibility with a fossil amine (FA) and a bio-based phenalkamine (PK) crosslinker was evaluated. Complementary to existing knowledge summarized before, the effects of diluents and crosslinker combinations on the properties of epoxy coatings are not uniquely known and depend on a balance between the reduction in viscosity, softening, and lubrication. Therefore, a comprehensive study is presented where the coating performance was related to the intrinsic mechanical properties for both fossil- or bio-based origin of diluents and crosslinkers. The present results indicate good opportunities for a transition of fossil-based into bio-based epoxy coating formulations with controllable performance.

## 2. Materials and Methods

### 2.1. Materials

The bisphenol A diglycidyl ether (DGEBA) epoxy resin was purchased from Resion Resin Technology (Moordrecht, The Netherlands) with the commercial name EP101. The reactive diluents with different functionalities were purchased from Merck (Darmstadt, Germany), including a grade of fossil-based monofunctional glydicyl ether (MGE), fossil-based di-functional glycidyl ether (DGE), and a bio-based tri-functional glycidyl ether (TGE) that was synthesized from glycerol. As an alternative reactive diluent, the epoxidized soybean oil (ESBO) was obtained from Merck (Darmstadt, Germany), with a molecular weight of 950 g/mol and epoxide equivalent of 230 g/mol. The latter indicates an average of 4.2 oxirane groups per molecule, therefore representing a multifunctional diluent with higher functionality than the previous diluents. The chemical structures and epoxy equivalent weight (EEW) for the epoxy resin and diluents are detailed in Table 1.

**Figure 1:**
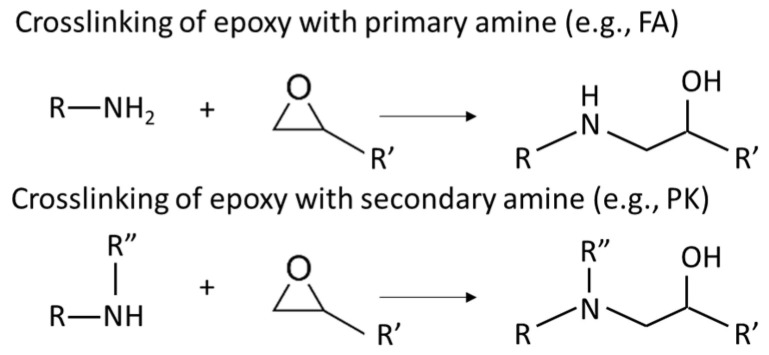
Main reaction mechanisms for crosslinking of an epoxy resin using ring-opening reaction in presence of primary or secondary amines.

Two types of crosslinkers with optimized composition for epoxy coatings were used with either fossil-based or bio-based origin. The fossil amine (FA) was a commercially available fast crosslinking cycloaliphatic amine containing a mixture of 3-aminomethyl-3,5,5-trimethylcyclohexylamine (30 to 50 wt.-%) and *m*-phenylene bis(methylamine) (10 to 30 wt.-%), with the trade name EP113 (Resion Resin Technology, Moordrecht, The Netherlands). The bio-based phenalkamine (PK) was obtained after a reaction between cardanol and 1,2-ethylenediamine and is commercially available under the trade name H811 (Anacarda, Wigan, UK). The chemical structures and amine hydrogen equivalent weight (AHEW) for crosslinkers are detailed in Table 2. The main reactions for the crosslinking of an epoxy resin with a primary amine (e.g., FA) or secondary amine (e.g., PK) are shown in Figure 1.

### 2.2. Coating Formulation and Application

The coating formulations were prepared by mixing the epoxy resin with different concentrations of MGE, DGE, TGE (1, 2, 3, 4, 5, 7.5 wt.-%), or ESBO diluent (10, 20, 30, 40 wt.-%), as well as a stoichiometric ratio of FA or PK crosslinker. The coating compositions were made by first stirring the epoxy resin with the diluent and subsequently adding the crosslinker after 10 min stirring time, followed by a 5 min stirring time in the presence of the crosslinker. A combination of diluents was applied for the resin mixtures of DGEBA + 30 wt.-% ESBO with TGE diluent (1, 2, 3, 4, 5, 7.5 wt.-%). The *EEW_mix_* of the epoxy resin mixtures (i.e., DGEBA + diluent) was calculated according to Formula (1) and the values are summarized in Table 3, giving an overview of the epoxy compositions included in the present testing series. The weight fraction of the DGEBA resin (*w_DGEBA_*) and diluent (*w_diluent_*) were determined on an analytical balance with 0.001 g accuracy. The weight of the added crosslinker (*w_PK/FA_*) per 10 g epoxy mixture was calculated from a 1:1 stoichiometric ratio between functional epoxy groups and amine crosslinker resulting from the respective *EEW_mix_* and AHEW values, according to Formula (2).

(1) $${EEW}_{mix}=\frac{w_{DGEBA}\;+\;\;w_{diluent}}{\frac{w_{DGEBA}}{{EEW}_{DGEBA}}\;\;\;+\;\;\frac{w_{diluent}}{{EEW}_{diluent}}\;}$$

(2) $$w_{PK/FA}\;\left(g\right)=\frac{AHEW}{{EEW}_{mix}}\;\cdot \;10\;(g)$$

The coatings were deposited via blade coating onto softwood beech substrates (10 cm × 10 cm × 5 cm), which were primarily planed and dried in a hot air oven overnight at 60 °C. The use of a constant blade speed at 5 mm/s resulted in a wet thickness of 70 µm and was verified with a coating thickness gauge indicating a 68 ± 2 µm dry thickness. The coatings were fully cured under controlled laboratory conditions of 25 °C and 60% relative humidity for one month before further testing. No thermal curing was applied in agreement with practical on-site application in the wood-coating industry. Similarly, films of the same thickness and compositions (further used for mechanical testing) were obtained after deposition and peeling off the coating from a non-sticky aluminum support.

### 2.3. Characterization Methods

The viscosity measurements were performed according to ADTM D2196 using a DV-III Ultra viscosimeter with spindle SC4-27RD (Brookfield Engineering, Hadamar-Steinbach, Germany) under a constant rotational shear rate of 100 rpm over a time of 1 min at a controlled temperature of 25 °C. The DSC measurements were performed on a DSC 3+ (Mettler Toledo, Columbus, OH, USA) on a liquid sample to follow the curing reaction as a function of temperature, or on a solid cured sample to determine the glass transition temperature *T_g_*. For the liquid coatings, a sample size of 4 mg was heated in hermetically sealed aluminum pans between 10 and 210 °C at 5 °C/min under a nitrogen atmosphere. For the solid samples, a sample size of 7 mg was heated during two heating cycles between 20 and 110 °C at 10 °C/min under a nitrogen flow. The thermal characteristics were determined from the second heating cycle.

The abrasive wear of coatings was evaluated via Taber testing according to ASTM D4060-10 using a circular rotary platform (Model 5130, Taber Industries, New York, NY, USA) with calibrated CS-10 abrasive wheels loaded under a 250 g or 500 g load and 72 rpm rotational speed. The tests ran over 1000 cycles and the weight loss was determined on an analytical balance with an accuracy of 0.001 g (Sartorius, Göttingen, Germany). The microhardness was measured according to ASTM D2240 [41] with a handheld Shore D micro-indenter with a standardized hardened steel tip of 30° and 0.1 mm tip radius. The gloss values were recorded according to ISO 2813 [42] with a micro-triglossmeter (BYK-Gardener Instruments, Geretsried, Germany) under a 60° incident light angle. The scratch resistance was evaluated according to ISO 4586-2 [43] with a sclerometer type 3092 (Elcometer, Aalen, Germany) by inserting a tungsten carbide tip of 0.75 mm radius under a load of 10 N or 20 N depending on the spring constant. The scratches were optically evaluated under a stereomicroscope MS12 (Leica, Wetzlar, Germany) at a magnification of 50×. The surface topography of the worn coatings was visualized with a VK-X3000 laser interferometer (Keyence, Mechelen, Belgium) at magnifications of 20× and 50×. The static water contact angles were measured according to ISO 19403-2 [44] after the deposition of 3 µL droplets of de-ionized water with an OCA 50 contact angle device (Dataphysics Instruments GmbH, Filderstadt, Germany) and fitting the droplet geometry using a tangent procedure that involved averaging the left and right contact angles. The water contact angles were determined 10 s after the deposition of the droplet and averaged over 10 measurements per sample with a standard deviation of ±2°.

The tensile testing of films was done on a universal testing machine (ProLine Z005, Zwick Roll, Haan, Germany) and impact testing was done on an Izod impact tester measuring the absorbed energy according to ASTM D256. The mechanical tests were repeated on 10 samples and reported as an average value for stress at break σ (MPa), strain at break ε (%), and impact strength (kJ/m^2^).

## 3. Results and Discussion

### 3.1. Coating Preparation and Curing Process

**Figure 2:**
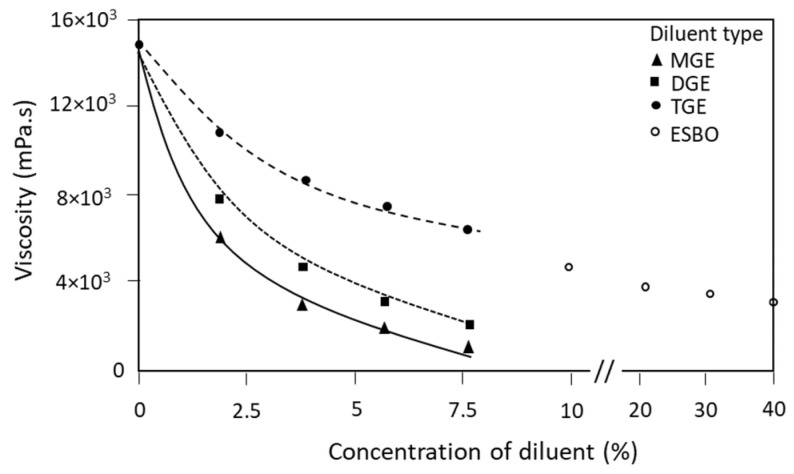
Variations in viscosity for epoxy resin with different types of diluents as a function of diluent concentration, lines are a visual aid to measuring points for illustrating trend.

The addition of diluents in an epoxy formulation is intended to lower the viscosity and enhance the processing during coating application. Therefore, results of the steady-state viscosity values for epoxy resin mixtures with different concentrations of diluents (MGE, DGE, and TGE at 0 to 7.5 wt.-% and ESBO at 10 to 40 wt.-%) before adding crosslinker are presented in Figure 2.

During the measurement, the viscosity stabilized after about 10 s and remained constant during the full recording time of 60 s, as no reaction between the epoxy resin and diluents happened in the absence of the crosslinker, while good pre-mixing of the resin and diluent had been achieved. The native epoxy resin inherently had the highest viscosity of 15,000 mPa·s, in agreement with the supplier’s data sheets, which posed problems in further application. The ability to reduce the viscosity in the presence of reactive diluents is demonstrated depending on the functionality and concentration of the diluent, where values can be reduced below 1000 mPa·s for practical coating application. The presence of reactive diluents favorably reduces and stabilizes the viscosity of epoxy resin such that it becomes systematically lower at higher concentrations. The viscosity of epoxy resin with an MGE diluent was the lowest, in agreement with the high flexibility of the linear polymer chain. It is known that monofunctional diluents are most efficient at reducing the viscosity of paints and coatings, as demonstrated for alkyds [45], but they may simultaneously reduce the crosslinking density and decrease the mechanical properties, as demonstrated further on for the present epoxy coatings. In agreement with other studies, an efficient reduction in viscosity of epoxy-reactive diluent mixtures was only observed after the addition of the first 5 wt.-% of glycidylether [46]. Therefore, a good balance between the processing and performance of epoxy coatings needs to be further identified. Although it has a slightly higher viscosity of epoxy resin with DGE diluent, it remains in a similar range as the MGE diluent owing to the linear polymer chains, in combination with the difunctional epoxide groups. The branched polymer structure of TGE evidently increased the viscosity due to its enhanced ability to produce molecular entanglements. For a more complex ESBO diluent, higher concentrations were needed to obtain a significant reduction in viscosity, in agreement with previous studies, where at least 50 wt.-% ESBO was needed to reduce the epoxy to the same viscosity [47]. Indeed, the viscosity of an epoxy resin decreases at higher concentrations of the reactive diluent, but it also strongly depends on the molecular weight of the diluent [48], which is obviously higher for ESBO compared with the glycidyl ethers. The reactivity of multifunctional diluents is more comparable with the base epoxy resin [49], and it is therefore expected that they less drastically affect the crosslinking density of the epoxy compared with MGE. For this reason, ESBO concentrations up to a maximum of 40 wt.-% were included in this investigation. In that case, however, a balance with the reduction in mechanical properties must be found. It can be concluded that the diluents with simple linear molecular configuration already suitably reduced viscosity at low concentrations, while the higher concentrations of more complex multifunctional diluents were efficient for viscosity reduction.

**Figure 3:**
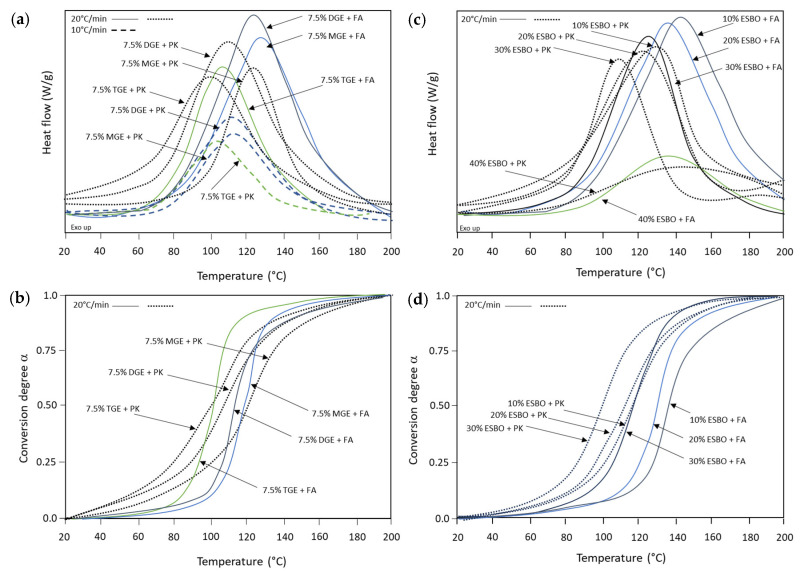
Non-isothermal DSC thermographs with exothermal reaction and calculated conversion degree from monitoring the crosslinking reaction of epoxy resin with different diluents and crosslinkers: (**a**,**b**) influence of diglycidyl ether diluents with different crosslinkers and (**c**,**d**) influence of vegetable oil with different crosslinkers.

The crosslinking of epoxy resin with different reactive diluents was followed by the exothermal peak during DSC analysis, after adding stoichiometric amounts of FA or PK crosslinker. Although reaction kinetics are traditionally studied using isothermal DSC analysis, the crosslinking of epoxy coatings was explicitly monitored in this study using non-isothermal data, as the optimum temperature ranges for FA and PK crosslinker agents may be different and can be more efficiently detected. A detail of the exothermal peak during heating of liquid coating samples between 20 and 200 °C is illustrated for coating compositions containing epoxy with 7.5 wt.-% MGE, TGE, and DGE (Figure 3a), or variable concentrations of ESBO (Figure 3c).

Depending on the reactive diluent, the crosslinking reaction was postponed for the epoxy coatings with MGE: although possessing the lowest viscosity and high molecular mobility, the low functionality limited the availability of the epoxy rings for ring-opening reactions. In parallel, the crosslinking of epoxy coatings with TGE diluent shifted to lower temperatures due to the higher reactivity of the trifunctional epoxy molecules, but the intensity of the curing reaction was lower in parallel with the higher viscosity and more difficult diffusion processes of reactive moieties. The DSC characteristics for diluents with FA crosslinker were evaluated at 10 °C/min and 20 °C/min curing rates, where the exothermal peak evidently became stronger and shifted toward higher temperatures at the higher heating rates, while the curing properties for different epoxy coating compositions were repeatable. For the ESBO diluent, the crosslinking shifted toward lower temperatures for concentrations up to 30 wt.-%, while the highest concentrations of 40 wt.-% did not yield favorable crosslinking. The latter may be explained through the steric hindrance caused by the long fatty acid molecules. The differences between FA and PK crosslinkers were clear for all epoxy compositions with a shift toward lower temperatures for PK: this is in line with the higher reactivity of PK at low temperatures, where the crosslinking may partially start at 30 °C. The higher reactivity of PK crosslinkers was noticed before [50], which can be attributed to the molecular structure with highly accessible amine groups and high reactivity of the hydroxyl group, enabling the curing in room temperature conditions. The accelerated crosslinking with PK proceeded faster and reached the maximum reactivity at a lower temperature, as is clearly noticed in the thermographs with shoulders at 30 to 50 °C, indicating the start of crosslinking. Meanwhile, the intensity of the exothermal peak for PK curing, corresponding to the total reaction heat (ΔH_R_), was lower in parallel with a slower crosslinking reaction that allowed for better control of heat dissipation.

The quantitative data were obtained after calculating the conversion degree α as a function of curing temperature (Figure 3b,d) and fitting the S-shape curves to an Arrhenius equation, which allowed for determining the characteristic heat and kinetic parameters for the curing process. The conversion degree was calculated from the non-isothermal DSC thermographs as the integrated exothermal heat at temperature T (ΔH_T_) relative to the total exothermal heat during the entire curing reaction (ΔH_R_), i.e., α = ΔH_T_/ΔH_R_, where both values of heat enthalpy were calculated via the integration of the heat flow curve over the appropriate temperature range. The reaction rate da/dt can be expressed as a generalized Equation (3) [51], assuming n^th^-order curing kinetics with a specific reaction rate constant *k*(*T*) at a given temperature T. These parameters can be calculated from experimental data according to known procedures in the literature based on the kinetic modeling of DSC data after fitting to a model that assumes an Arrhenius-type expression of *k*(*T*) following Equation (4), with gas constant R = 8.314 J/(mol K) [34]. The parameters from thermal analysis and kinetic modeling for the curing of a selection of coatings are reported in Table 4, including the total reaction heat (ΔH_R_), peak temperature (*T_p_*), activation energy (*E_a_*), kinetic factor *A*, and reaction order *n*.

(3) $$\frac{d\alpha }{dt}=k\left(T\right){\left(1-a\right)}^{n}$$

(4) $$k\left(T\right)=A\;e^{-\frac{E_{a}}{RT}}$$

The activation energy *E_a_* represents an input value required as a barrier to initiate the crosslinking. A higher *E_a_* would generally postpone the crosslinking reaction; however, the reaction rate after initiation also strongly depends on the molecular structure of the reactants. For regular epoxy/amine systems, a value for activation energy *E_a_* = 50 to 70 kJ/mol is reported [52], which is in a similar range as that calculated in our study for the FA crosslinkers. As demonstrated in previous studies, the reactive diluent (e.g., diglycidyl aniline) decreases both the activation energy and the cure kinetic parameters [53]. Depending on the use of diluents and/or a crosslinker, the epoxy system with low *E_a_* enables an easy crosslinking toward a high degree of conversion. Depending on selected diluents, the *E_a_* decreased and A increased compared with a pure epoxy coating due to the lower viscosity and reactivity of the diluent. It can be reasoned that crosslinking was easier for the more flexible polymer chains and accessible functional groups with, consequently, a lower *E_a_*, but this had to be balanced against the mobility of reactive groups and the viscosity of the medium. For the DGE diluent, the linear structure and accessibility of terminal epoxy groups favorably increased the reactivity of the system, resulting in a low *E_a_*. For the MGE diluent, the high *E_a_* may relate to the monofunctional epoxy groups and more frequent termination of the crosslinking reaction. It was indeed confirmed for other types of difunctional diluents that the activation energy for the curing process reduced most favorably [54]. For the TGE diluent, the high *E_a_* may have resulted from the formation of a dense three-dimensional molecular network from the beginning of the curing reaction, which may have hindered the reaction kinetics. Other studies showed the fastest curing and coating drying times for epoxy resins with trifunctional diluents [55]. Alternatively, the reaction rate A corresponds to the chance for the collision of reactive groups, leading to a favorable reaction due to the higher functionality of the diluent. Moreover, it was previously demonstrated that the reaction rate also depends on the polarity of the medium, and the crosslinking traditionally follows autocatalytic properties through the presence of -OH groups [56]. However, only minor variations in reaction kinetics for diluents were found and larger differences were observed for the selection of different crosslinkers. The low *E_a_* and A for PK crosslinkers were mainly due to the high reactivity and intrinsic molecular properties of the PK crosslinker versus FA crosslinker. In particular, the *E_a_* for PK-epoxy with diluents was within the lower range or incidentally slightly below the ones reported for traditional epoxy/amine curing. The crosslinking kinetics in the presence of ESBO diluent are more complex and were also documented before [57], where the high *E_a_* and low kinetic factors were reported in parallel with the relatively high viscosity and more complex entanglements of the long fatty acid chains hindering the diffusion of reactive moieties. Irrespective of the used FA or PK crosslinkers, the kinetics evidently followed first-order kinetics with *n* = 0.9 to 1.0, as expected for amine crosslinking.

**Figure 4:**
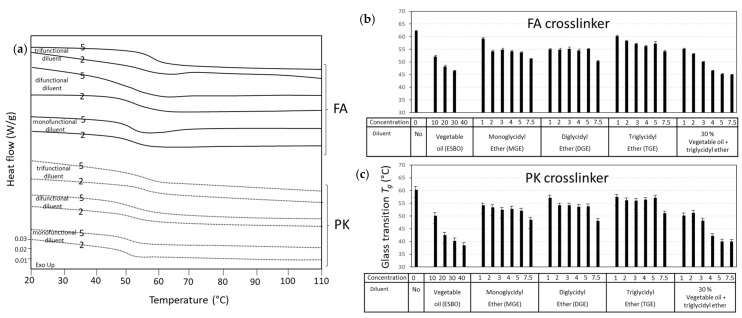
Glass transition temperature *T_g_* of crosslinked epoxy resins with different diluents and crosslinkers: (**a**) details from DSC thermographs of some compositions, (**b**) *T_g_* values for FA-epoxy and different diluents, and (**c**) *T_g_* values for PK-epoxy and different diluents.

The *T_g_* values for fully cured coatings were determined from the second DSC heating scan, where some exemplary graphs (Figure 4a) and a summary of the values are presented for FA-epoxy coatings (Figure 4b) and PK-epoxy coatings (Figure 4c). The main variations in *T_g_* depending on the crosslinker and reactivity of the amine groups are expected to depend on the crosslinking density of epoxy [58]. The *T_g_* relates to the mobility of the molecular segments in the polymer chain and, therefore, depends both on the crosslinking density and chain length in the opposite way: as the crosslinking density increases and the chain length of molecular segments in between the crosslinking points decreases, the polymer becomes more rigid, and *T_g_* increases. Therefore, both the functionality and chain length of diluents influence a shift in *T_g_*. The *T_g_* for epoxy coatings without diluents is highest, as it is generally known that diluents cause a reduction in *T_g_*: owing to the molecular structure of diluents, a more flexible polymer segment is introduced relatively to the stiff aromatic structure of the epoxy resin. It was previously demonstrated that *T_g_* decreases at higher diluent concentrations due to the higher chain flexibility [59]. On the other hand, the diluents with higher functionality may increase the crosslinking density and simultaneously reduce the molecular mobility. Therefore, the balance between both physical mechanisms may introduce only very slight variations in *T_g_*. However, there was a clear trend showing that *T_g_* increased for the diluents with higher functionality and tended to stabilize or slightly decrease with higher diluent concentrations. For the vegetable oil diluent, the low *T_g_* values were related to the relatively large molecular segments of fatty acid side chains with high molecular mobility. Alternatively, the PK crosslinker led to a lower *T_g_* for the pure epoxy resin owing to the more flexible side chain of the phenalkamine molecule, while the influence in the presence of diluents was more complex and marginally lower in comparison with the FA crosslinker.

### 3.2. Tribological Coating Performance

**Figure 5:**
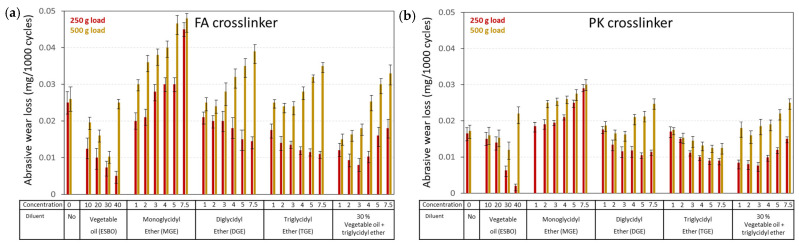
Wear rates under low load (250 g) and high load (500 g) for epoxy coatings with different diluents and crosslinkers for (**a**) FA-epoxy and (**b**) PK-epoxy.

The abrasive wear was determined as the weight loss after Taber testing under a low load (250 g) and high load (500 g) and is represented for coatings of FA-epoxy (Figure 5a) and PK-epoxy (Figure 5b). The wear loss was evidently higher under a high load; however, the increase in abrasive wear with applied load may not be linear due to visco-elastic properties and deformation of the epoxy coating. Different trends between low- and high-loading conditions were observed due to frequent overload conditions in the latter case. Similar trends were observed for various diluents and concentrations in the FA-epoxy and PK-epoxy coatings, with a tendency for lower wear rates and less frequent overload conditions under high loads for the bio-based PK-epoxy relative to the fossil-based FA-epoxy. These trends are further explained in relation to the mechanical properties of the epoxy coatings. Depending on the type and concentration of used diluent, wear rates were lower compared with the pure epoxy coating. The lowest wear rates obviously occurred for vegetable oil diluents due to the lubricating properties of the oil, as mainly illustrated at the highest concentrations of 40 wt.-%, where residual free oil molecules should be present owing to the unsuccessful crosslinking reactions at high concentrations, as demonstrated before. The functionality of the glycidyl ether diluents predominantly influenced the wear properties, where the higher functionalities in a series of MGE < DGE < TGE gradually reduced the wear rates, as expected due to a higher crosslinking density for diluents with high functionality. The compatibility between vegetable oil and TGE diluent for stabilizing wear rates was demonstrated up to limited concentrations of the ether diluent.

**Figure 6:**
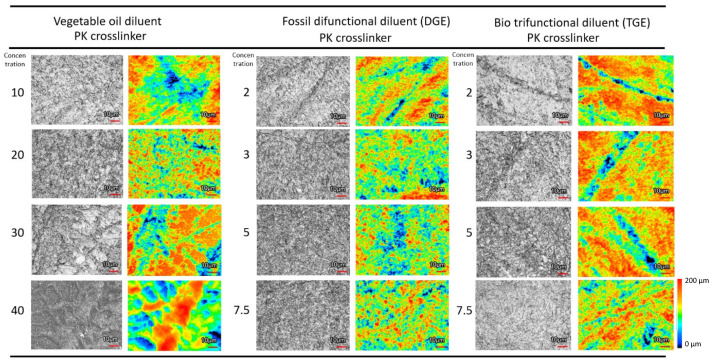
Microscopic evaluation of the wear tracks indicating influence of diluents for some PK-epoxy coatings, including laser intensity image (greyscale image) and height map (color picture).

**Figure 7:**
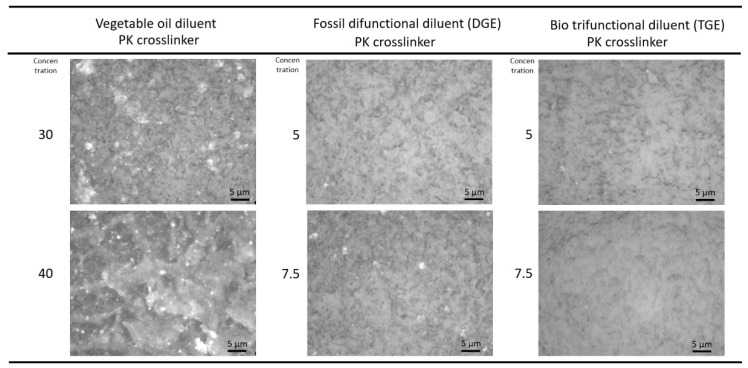
Detailed optical microscopy of wear tracks indicating influence of diluents for some PK-epoxy coatings.

The morphology of the wear tracks (after wear at the highest load) was evaluated through laser interferometric microscopy and three-dimensional surface topography (Figure 6), illustrating the effect of various types and concentrations of diluents. The wear tracks are shown only for PK-epoxy coatings, but similar trends and conclusions on the influence of diluents can be drawn for FA-epoxy coatings (here, PK-epoxy coatings are presented as they provided the lowest wear). The surface morphology for worn coatings with vegetable oil diluents showed highly deformed surface structures, as mainly observed at the highest concentrations of 40 wt.-% oil. This was likely due to the presence of free oil molecules that provided lubricating properties, which is in line with the relatively low wear rates and presence of an almost liquid surface film. The coatings with the DGE diluent were more severely worn, with significant wear scars in the bulk of the coating, as represented by a more brittle aspect, in comparison with the epoxy coatings with TGE diluents, which showed more superficial wear at the surface of the coating and only some deeper local grooves. The smooth top surface of coatings with TGE diluent is in line with the low wear rates. The more detailed optical microscopy images of the worn surfaces (Figure 7) support the observations above, with most irregular surfaces for vegetable oil diluents, strongly worn surfaces for DGE diluents, and smooth surfaces for TGE diluents.

### 3.3. Mechanical Coating Performance

**Figure 8:**
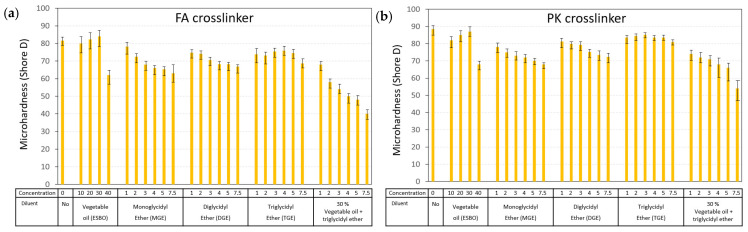
Microhardness for epoxy coatings with different diluents and crosslinkers for (**a**) FA-epoxy coatings and (**b**) PK-epoxy coatings.

**Figure 9:**
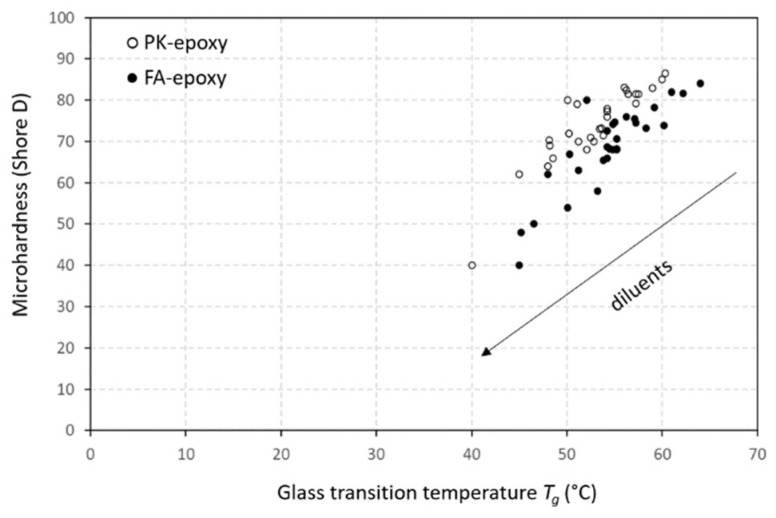
Relationship between microhardness measurements and glass transition temperature for epoxy coatings with different diluents and crosslinkers.

The coating microhardness is a primary indicator of mechanical resistance and is related to the resistance against plastic deformation. The influence of different types and concentrations of diluents on the microhardness followed consistent trends after the crosslinking of FA-epoxy (Figure 8a) or PK-epoxy (Figure 8b). The crosslinking with PK obviously provided coatings with a higher hardness relative to the FA crosslinkers for all diluent compositions. The higher hardness for PK-epoxy coatings is in line with previous studies without the use of diluents [10], where it was related to the higher degree of crosslinking for PK-epoxy relative to the FA-epoxy coatings. The high hardness and tensile and flexural strengths of PK-epoxy compared with conventional FA-epoxy were also confirmed in other studies, depending on the selection of the phenalkamine [60], where the crosslinking density of the PK-epoxy coatings was higher compared with the FA-epoxy coatings. The microhardness of coatings clearly depended on the functionality of the diluent and expected crosslinking density, with the relatively high functionality of vegetable oil diluents leading to a high hardness. Alternatively, the increasing functionality for the MGE < DGE < TGE diluents resulted in progressively increasing microhardness for the same concentrations of diluent. The relationships between microhardness and *T_g_* are demonstrated (Figure 9), where both parameters were inherently related through the restricted molecular mobility for coatings with a high *T_g_* and high hardness. Thus, the coating performance was uniquely determined by the intrinsic mechanical parameters.

**Figure 10:**
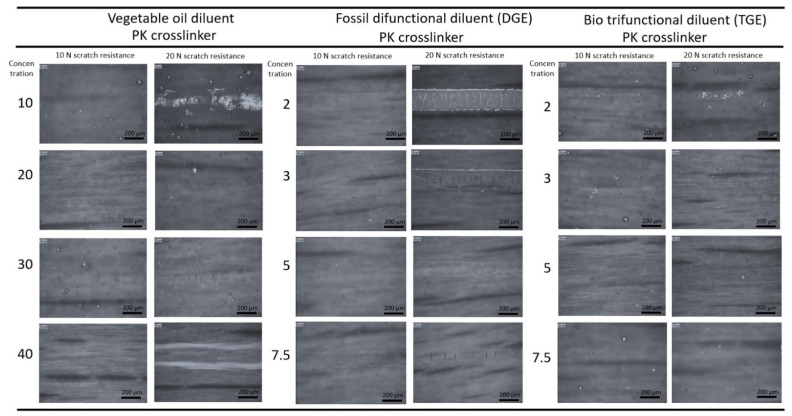
Optical microscopy illustrating scratch resistance of PK-epoxy coatings with different diluents after scratching under 10 and 20 N normal loads.

The scratch resistance under 10 N and 20 N was evaluated using optical microscopy for coatings with different diluents and the PK crosslinker (Figure 10). The scratch resistance of the FA-epoxy coatings (not presented) was worse than for PK-epoxy coatings, showing breakthrough under the 10 N load due to the lower microhardness. Depending on the diluent, the coatings with vegetable oils did not show scratch damage under 10 N and they had a strongly deformed scratching track for 40 wt.-% vegetable oil, representing ductile deformation. The scratch resistance improved for diluents in the series MGE < DGE < TGE due to the increasing functionality. The coatings with TFE diluents showed the highest scratch resistance without surface damage for concentrations of 2 to 7.5 wt.-%. It is known that resistance against scratch damage depends on the tensile strength and compressive yield stress of the epoxy [61], which directly relates to the crosslinking density. The scratch resistance is known to be enhanced by the higher crosslinking density, which can be enhanced by tuning the processing conditions or using supplementary additives [62]. Given the higher *T_g_* for diluents with a higher functionality and PK versus FA crosslinker, the scratch resistance was expected to increase [63]. Moreover, delamination of the coating after scratching was not observed, showing good interface adhesion for all cases.

**Figure 11:**
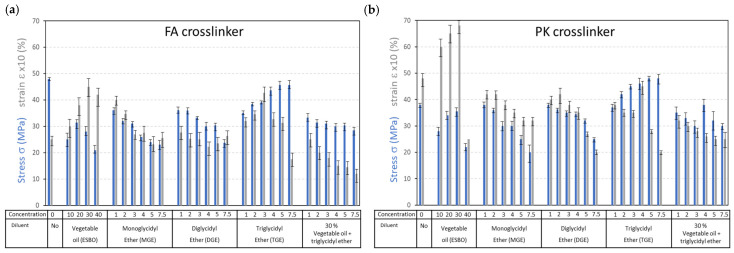
Mechanical test results with overview of stress at break (blue bars) and strain at break (grey bars) of epoxy compositions with different diluents and crosslinkers for (**a**) FA-epoxy and (**b**) PK-epoxy.

The mechanical test results for the maximum tensile stress (σ) and strain at break (ε) were determined from standard tensile testing for FA-epoxy (Figure 11a) and PK-epoxy (Figure 11b), yielding higher elongation and lower strength for PK-epoxy compared with FA-epoxy. Therefore, it can be noticed that the PK-epoxy behaved as a ductile material and the FA-epoxy was more brittle. The main trend showing higher elongation for PK-epoxy remained in the presence of diluents, while the elongation decreased a little for diluents with higher functionality, i.e., MGE > DGE > TGE. The higher ductility for epoxy resins with di- and multifunctional diluents was also demonstrated in previous studies [40]. The increase in ductility and toughness of epoxy via the selection of a suitable diluent and crosslinker is an alternative to the formulation of epoxy-based nanocomposites with improved ductility [64]. The elongation of epoxy with vegetable oil diluents becomes extremely high and represents the high flexibility that can be introduced through the combination of long side chains in both the PK crosslinker and fatty acid molecules. The tensile strength for the epoxy with higher functionality increased as an illustration of the higher degree of crosslinking with DGE and TGE while maintaining good flexibility for PK-epoxy and becoming brittle for FA-epoxy.

**Figure 12:**
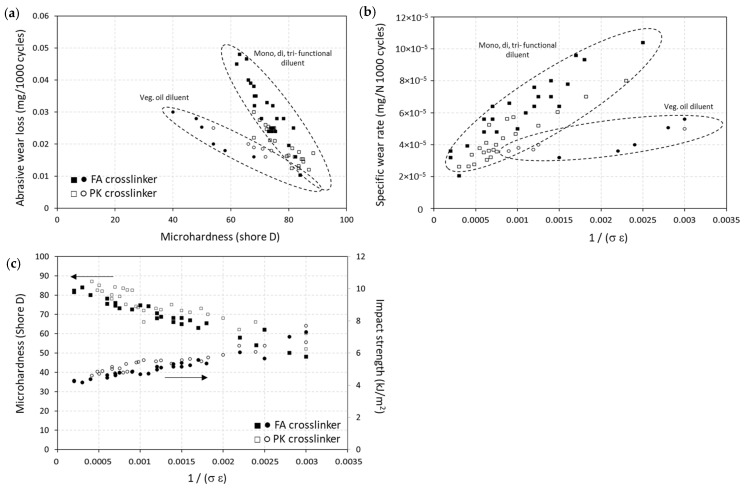
Relationships between intrinsic mechanical properties and coating performance: (**a**) abrasive wear against microhardness, (**b**) abrasive wear against ductility (Lancester plot), and (**c**) microhardness and impact strength against ductility.

In summary, the relationships between the intrinsic mechanical properties and tribological performance of the epoxy coatings are presented in Figure 12, including the abrasive wear loss, microhardness, and impact resistance, in relation to tensile stress (σ) and strain at break (ε). The wear loss was related to the microhardness of the epoxy coatings (Figure 12a), with better wear resistance for coatings with a high microhardness. This trend was uniquely confirmed for the MGE, DGE, and TGE diluents for both FA-epoxy and PK-epoxy. The trend for vegetable oil diluents followed a higher level of wear resistance for coatings with comparable hardness, illustrating the additional lubricating effect of the vegetable oil diluent. The Lancester plot representing specific wear rates against ductility (Figure 12b) was applied well for the present epoxy coatings, again indicating the additional lubricating properties of the vegetable oils. The good fit of the present experimental data and correspondence to known material models confirmed that the coating properties were well controlled through variations in the composition and related crosslinking conditions. The microhardness and impact resistance were also related to the intrinsic mechanical properties of the epoxy coatings with various diluent concentrations and crosslinker types (Figure 12c), where values for the hard and brittle coatings (FA-epoxy) and more ductile coatings (PK-epoxy) overlapped for the same hardness value. The slightly higher impact strength for PK-epoxy relative to the FA-epoxy coatings was noticed, in agreement with its higher flexibility and ductility. Similarly, an increase in impact strength and ductility of epoxy resins with higher diluent concentrations was demonstrated before [55]. The impact resistance for coatings with difunctional diluents (DGE) was slightly higher than other diluents (MGE, TGE) in relation to the other mechanical properties, as demonstrated in this study. Depending on the selected diluent in combination with a crosslinker, it was observed that the wear properties of coatings were determined by the intrinsic mechanical properties.

### 3.4. Coating Surface Properties

**Figure 13:**
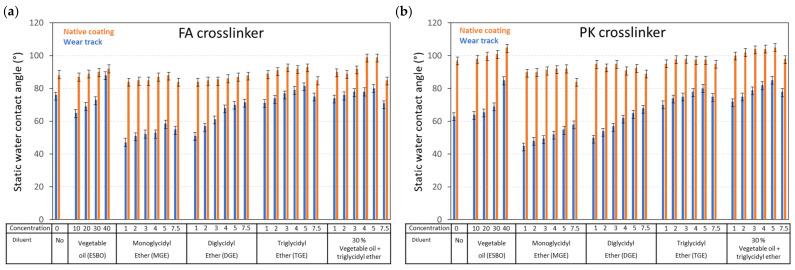
Coating surface properties for epoxy coatings with different diluents and crosslinkers, including static water contact angles before and after wear, for (**a**) FA-epoxy and (**b**) PK-epoxy (statistical variation ±3°).

The hydrophobicity of epoxy coatings was characterized through static water contact angles (Figure 13), which were determined as steady-state values after 10 sec of stabilization time. The water contact angles were determined on coatings before and after wear, identifying the effect of wear on long-term hydrophobic protection. The reference uncoated wood substrate showed unstable water contact angles, with immediate absorption of the water, while protection and stabilization against water ingress were obtained for coated wood. The role of FA versus PK crosslinker for the pure epoxy coatings (no diluents) was determined before [10], where the hydrophobicity of PK was not directly identified on the native coatings and it was only expressed in the wear track, as expected from the long hydrophobic polymer side chains. The latter is explained in terms of the relatively higher degree of crosslinking of PK-epoxy and unfavorable orientation of the hydrophobic groups in the bulk of the coating rather than being exposed at the surface. The hydrophobicity in the presence of vegetable oil diluents was very high due to the presence of non-crosslinked oil diluents, as confirmed before by the low *T_g_* values. The exposure of free oil molecules at the surface after wear evidently provided the highest hydrophobicity. For the other diluents, the hydrophobicity increased with higher concentration and higher functionality of the diluent with consistent trends for both crosslinker types and constantly some lower contact angles on PK-epoxy relative to FA-epoxy. Some higher contact angles on worn coatings of PK-epoxy compared with FA-epoxy were observed due to both chemical and topographical changes to the coating surface after wear. In particular, the polarity of C-O bonds or long aliphatic tails in the polymer chain of diluents was expressed in the high coating hydrophobicity.

**Figure 14:**
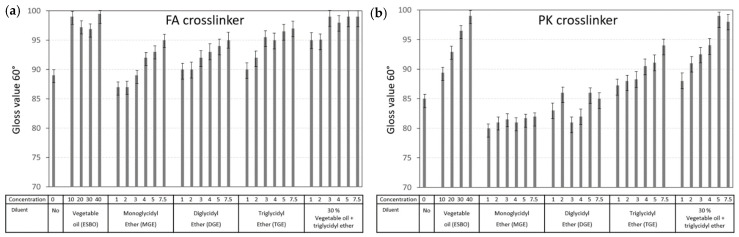
Coating surface properties for epoxy coatings with different diluents and crosslinkers, including gloss values measured under 60° incident light, for (**a**) FA-epoxy and (**b**) PK-epoxy.

**Figure 15:**
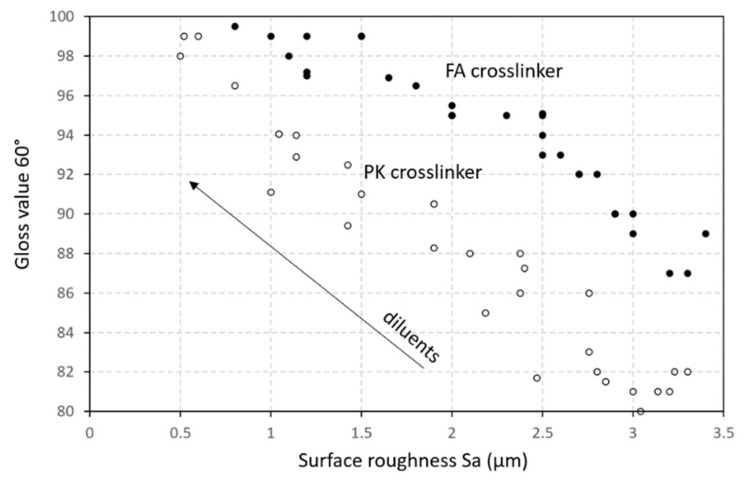
Relationships between surface properties of gloss versus surface roughness Sa for FA-epoxy and PK-epoxy coatings.

The surface gloss for epoxy coatings with various diluents was compared after crosslinking with FA and PK (Figure 14). Relative to a pure epoxy coating (no diluents), the gloss improved after mixing with diluents in increasing concentrations. Mainly in the presence of the vegetable oil diluent, an amount of free non-crosslinked fatty acids may migrate to the surface and aid in the formation of a glossy surface. For other diluents, the lower viscosity and better flow properties of the liquid coating may result in a smoother surface and consequently higher gloss. The gloss for coatings with a PK crosslinker was somewhat lower compared with the FA crosslinkers due to the dark brown color. The variations in gloss can be explained in relation to the measurements of surface roughness Sa on the native coatings (Figure 15), where a good relationship was observed between both parameters for both the FA-epoxy and PK-epoxy coatings: the smooth coating surfaces in the presence of diluents were clearly related to a higher gloss. The differences in intrinsic color between FA-epoxy and PK-epoxy coatings may indeed explain the distinctions in optical properties between both crosslinkers. In conclusion, the lower surface roughness of coatings with diluents contributed to the higher gloss owing to the better flow properties and higher surface homogeneity (e.g., fewer flow defects at lower viscosity).

## 4. Conclusions

The use of reactive diluents with different functionalities and bio-based or fossil-based origins in combination with a fossil-based and bio-based crosslinker provides a toolbox to optimize the processing conditions and performances of epoxy coatings. The viscosity reduction was related to a combination of flexibility and functionality of the reactive diluent, where lower concentrations of the glycidyl ether diluents were more efficient compared with the higher concentrations of the vegetable oil diluents. The reduction in viscosity for mono- and di-functional diluent was within comparable ranges for fluent coating processing, where a more branched structure of the bio-based trifunctional diluent evidently increased the viscosity due to the enhanced ability for molecular entanglements. The curing kinetics for epoxy with difunctional diluent showed the lowest activation energy and highest reaction rates due to a favorable combination of accessibility and reactivity of the epoxy groups, while the kinetics were slowed down for a bio-based trifunctional diluent in parallel with the higher viscosity of the reaction mixture. All types of diluents induced first-order reaction kinetics, irrespective of their source. Although the variations in reaction kinetics for different diluents were minor, the use of a bio-based phenalkamine crosslinker had more of an effect on accelerating the crosslinking, mainly at lower temperatures.

The mechanical coating properties were strongly related to the glass transition temperature of the respective epoxy formulations, which was lowered in the presence of the diluents and phenalkamine crosslinkers. The latter indeed introduced more ductile properties reflected in their mechanical properties. The reduction in abrasive wear for trifunctional diluents and phenalkamine crosslinkers was the highest in parallel with the high microhardness and scratch resistance. Any further reduction in abrasive wear in the presence of vegetable oil diluents was due to additional lubrication of the diluents rather than related to the intrinsic mechanical parameters. In particular, the long-term protection of the coating was retained by high hydrophobicity, which was evidently the highest in the presence of vegetable oil diluents, but also remained high for the trifunctional bio-based diluent.

Overall, good relationships could be drawn between the intrinsic mechanical properties and coating properties for glycidyl ether diluents of different functionalities and two types of amine crosslinkers, independent of their bio-based or fossil-based origin. As such, the differences in the intrinsic chemical structure of the latter allow for tuning the coating performance and select new formulations with higher bio-based content and appropriate properties.

## Figures and Tables

**Table 1:** Chemical structures and characteristics of resin and diluents for epoxy coatings.

Product Type	Chemical Formula	EEW	CAS
DGEBA epoxy resin	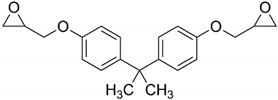 Bisphenol A diglycidyl ether	200 g/mol	1675-54-3
Fossil-based monofunctional diluent (MGE)	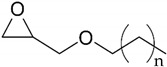 Monoglycidyl ether of C12-C14 fatty alcohol	250 g/mol	68609-97-2
Fossil-based difunctional diluent (DGE)	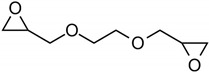 Ethylene glycol diglycidyl ether	142 g/mol	2224-15-9
Bio-based trifunctional diluent (TGE)	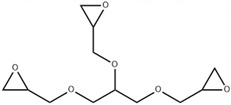 Glycerol triglycidyl ether	154 g/mol	13236-02-7
Bio-based multifunctional diluent	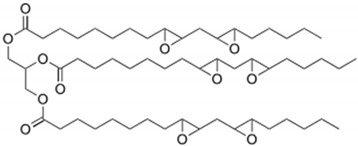 Epoxidized soybean oil (ESBO)	230 g/mol	8013-07-8

**Table 2:** Chemical structures and characteristics of fossil-based amine (FA) and bio-based phenalkamine (PK) crosslinkers for epoxy coatings.

Product Type	Chemical Formula	EEW	CAS
Fossil-based amine (FA)	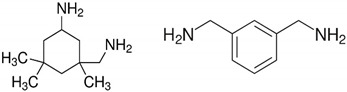	100 g/mol	2855-13-2 + 1477-55-0
Bio-based phenalkamine (PK)	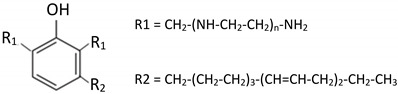	125 g/mol	868765-93-9

**Table 3:** Overview of testing matrix for epoxy coating formulations with different types and concentrations of diluents.

Diluent Type and Concentration (wt.-%)	MGE	DGE	TGE	30ESBO + TGE	Diluent Type and Concentration (wt.-%)	ESBO
	EEW Value (g/mol)					EEW Value (g/mol)
0	200			208.1		
1	203.7	192.8	194.7	201.7	10	202.6
2	206.9	187.3	190.5	196.6	20	205.3
3	209.6	182.8	187.1	192.4	30	208.1
4	212.1	179.0	184.3	189.1	40	211.0
5	214.3	176.0	181.9	186.3		
7.5	218.8	170.2	177.3	180.8		
	Amount (g) of FA per 10 g DGEBA mixed with x g diluent					Amount (g) of FA per 10 g DGEBA mixed with x g diluent
0	5			4.81		
1	4.91	5.19	5.14	4.95	10	4.93
2	4.83	5.34	5.25	5.08	20	4.87
3	4.77	5.47	5.35	5.19	30	4.80
4	4.71	5.58	5.43	5.28	40	4.73
5	4.66	5.68	5.50	5.36		
7.5	4.57	5.87	5.64	5.52		
	Amount (g) of PK per 10 g DGEBA mixed with x g diluent					Amount (g) of PK per 10 g DGEBA mixed with x g diluent
0	6.25			6.00		
1	6.13	6.48	6.42	6.20	10	6.17
2	6.04	6.68	6.56	6.35	20	6.10
3	5.96	6.84	6.68	6.49	30	6.00
4	5.89	6.98	6.78	6.60	40	5.92
5	5.83	7.10	6.87	6.71		
7.5	5.71	7.34	7.05	6.91		

**Table 4:** Thermal and kinetic factors for curing of epoxy coatings with a selection of diluents and crosslinkers.

	FA-Epoxy					PK-Epoxy				
	D*H*_R_ (J/g)	*T_p_* (°C)	*E_a_* (kJ/mol)	A (min^−1^)	n (-)	D*H*_R_ (J/g)	*T_p_* (°C)	*E_a_* (kJ/mol)	*A* (min^−1^)	*n* (-)
DGEBA	295	128	64.2	2.5 × 10^6^	0.999	281	132	52.3	2.8 × 10^6^	0.995
DGEBA + 2 wt.-% MGE	270	127	50.3	1.6 × 10^6^	0.998	228	121	38.3	0.9 × 10^6^	0.998
DGEBA + 5 wt.-% MGE	265	126	49.8	1.5 × 10^6^	0.998	212	122	40.2	1.0 × 10^6^	0.996
DGEBA + 2 wt.-% DGE	310	122	46.2	2.1 × 10^6^	0.975	254	110	35.2	1.3 × 10^6^	0.998
DGEBA + 5 wt.-% DGE	286	122	47.5	2.2 × 10^6^	0.988	232	112	39.5	1.2 × 10^6^	0.986
DGEBA + 2 wt.-% TGE	232	106	56.2	1.1 × 10^6^	0.993	215	99.5	42.2	0.7 × 10^6^	0.999
DGEBA + 5 wt.-% TGE	245	108	58.2	1.0 × 10^6^	0.998	205	106	45.1	0.6 × 10^6^	0.991
DGEBA + 20 wt.-% ESBO	194	133	83.4	1.3 × 10^6^	0.998	165	128	76.8	0.9 × 10^6^	0.990
DGEBA + 30 wt.-% ESBO	185	141	84.2	0.9 × 10^6^	0.997	159	122	80.8	0.7 × 10^6^	0.994

## Data Availability

The data presented in this study are available on request from the corresponding author. The data are not publicly available due to potential commercial interest.
